# Clinical and ultrasonographic features of medullary thyroid microcarcinomas compared with papillary thyroid microcarcinomas: a retrospective analysis

**DOI:** 10.1186/s12880-020-00444-9

**Published:** 2020-05-14

**Authors:** Xiaoyu Li, Wei Zhou, Weiwei Zhan

**Affiliations:** Ultrasound Department, Rui Jin Hospital, Shanghai Jiao Tong University School of Medicine, 197 Ruijin Er Rd, Shanghai, 200025 China

**Keywords:** Medullary thyroid carcinoma, Papillary thyroid carcinoma, Ultrasound

## Abstract

**Background:**

To identify the sonographic features that help to differentiate medullary thyroid microcarcinomas (MTMCs) from papillary thyroid microcarcinomas (PTMCs).

**Methods:**

A total of 46 MTMCs in 41 patients and 136 PTMCs in 104 patients that were proven by surgery and pathology were included in the study. Patient age and nodule size were analyzed by independent sample t-tests, and sex, multiplicity and cervical lymph node metastases were analyzed by χ^2^ or Fisher’s exact tests. Univariate analysis and multivariate logistic regression analysis were performed on the sonographic features of thyroid nodules, including location, shape, boundary, margin, peripheral halo ring, echogenicity, composition, calcifications and vascularization.

**Results:**

Compared with the corresponding number of patients with PTMCs, more MTMC patients had cervical lymph node metastases (*P* = 0.040). There were no significant differences in age, sex, nodule size, multiplicity, location, boundary, margin, peripheral halo ring, echogenicity or microcalcifications between MTMCs and PTMCs (*P* > 0.05 for all). However, significant differences were found in shape (*P* = 0.000), composition (*P* = 0.032), macrocalcifications (*P* = 0.004) and vascularity (*P* = 0.000) between the two groups.

**Conclusions:**

There were some overlapping sonographic features between MTMCs and PTMCs. However, MTMCs tended to have a > 50% solid composition, be ovoid to round nodules with macrocalcifications and be hypervascular. Cervical lymph node metastases were more common in MTMC patients.

## Background

Due to the wide use of ultrasound (US), the detection rate of small thyroid nodules, especially nodules less than 10 mm, has increased [1, 2]. At present, many controversies remain regarding the treatment of these small thyroid nodules with malignant features. Papillary thyroid microcarcinomas (PTMCs) are defined as papillary thyroid carcinomas (PTCs) less than 10 mm in diameter with a good prognosis. The American Thyroid Association (ATA) guidelines suggest that active surveillance could be considered in some PTMC patients. This strategy is not applied to medullary thyroid microcarcinomas (MTMCs), although their size is also smaller than 10 mm. Medullary thyroid carcinomas (MTCs) are derived from thyroid follicular C cells, account for 3–4% of all thyroid malignancies, and have a high recurrence rate and a poor prognosis [3]. MTCs are not sensitive to radiotherapy or chemotherapy. The 10-year mortality rate of MTCs is as high as 13.5–38% [4–6]. Early diagnosis and treatment are critical to improving disease-free survival and reducing the mortality rate of MTCs. Therefore, it is very important to differentiate PTMCs from MTMCs.

Previous studies on the ultrasonographic features of small thyroid nodules mostly focused on PTMC, which usually present as a solid and hyperechoic nodule with a taller-than-wide shape, an irregular margin and microcalcifications. However, due to the diversified sonographic manifestations and low incidence of MTMCs, its sonographic features have been reported in few studies with a limited number of cases. To the best of our knowledge, there has been only one report about the sonographic appearances of seven MTMCs that were compared with PTMCs [7]. To expand on these findings, this study aimed to evaluate the ultrasonographic characteristics of MTMCs, which may be useful to identifying MTMCs and provide useful information for the differential diagnosis of MTMCs and PTMCs.

## Methods

### Patients

This retrospective study was approved by the Ethics Committee of the Ruijin Hospital, Shanghai Jiao Tong University School of Medicine, along with the waiver of consent to participate. A systematic review consisted of MTMCs diagnosed from January 2008 to April 2017. A total of 41 patients with 46 MTMCs, which were surgically confirmed, were included in this study. Of the 41 patients, 35 patients (85.37%) had a sporadic type of MTC, 5 patients (12.20%) presented with multiple endocrine neoplasia (MEN) type II A, and one patient (2.43%) presented with MEN type II B. As a control group, 104 patients with 136 PTMCs confirmed by postoperative pathology from January to June 2015 were included in this study.

### Sonography imaging analysis

All patients were evaluated by US. The US examinations were performed with Philips IU22 (New York), GE LOGIQ E9 (New York), or MyLab 90 (Italy) devices equipped with a 5–12 MHz linear array transducer. All examinations were performed by two radiologists with more than 10 years of experience in thyroid imaging.

All thyroid nodules were evaluated in the transverse and longitudinal planes by grayscale and color Doppler ultrasonography. The sonographic features of the nodules were carefully evaluated, including location, shape, boundary, margin, peripheral halo ring, echogenicity, composition, calcifications, and vascularization. The cervical lymph nodes were also evaluated. All US images were retrospectively analyzed by the same radiologist who was blinded to the pathological results.

The location was determined as the upper or lower poles. Shape was assessed as ovoid to round, taller-than-wide or irregular. The boundary was classified as clear or unclear. Margin was divided into smooth, spiculated and poorly defined. A peripheral halo ring was defined as absent or present. Compared with the adjacent thyroid tissue, the echogenicity was categorized as markedly hypoechoic, hypoechoic, hyperechoic or isoechoic. According to the proportion of solid components, the composition was classified as solid or > 50% solid. Calcifications were recorded as absent or present. When calcifications were observed, they were categorized into microcalcifications (with diameters < 2 mm) and macrocalcifications (with diameters ≥2 mm or coexisting with microcalcifications). The nodule was considered hypervascular when the vascularity was similar to or greater than that of the surrounding thyroid tissue [8]. The remaining nodules were regarded as avascular.

### Data and statistical analyses

Statistical analysis was performed using the SPSS 20.0 software package (SPSS, Chicago, IL). Statistical significance was set as *P* < 0.05.

The independent-sample t-test was used to analyze age and size. Categorical variables, including sex, lymph node metastases, location, composition, shape, margin, boundary, echogenicity, calcifications, and vascularization, were analyzed by the χ^2^ test or Fisher’s exact test. The variables with statistical significance were included in the multivariate regression model. The B value, Wals χ^2^, ratio (OR) and 95% CI were recorded.

## Results

### Clinical characteristics of MTMCs and PTMCs

The clinical characteristics of the patients are summarized in Table 1. There were no significant differences in age (41.78 ± 16.39 years vs 44.07 ± 10.43 years, *P* = 0.323), sex (female: male ratio, 23:18 vs 70:34; *P* = 0.205) or tumor size (6.30 ± 2.27 mm vs 6.06 ± 2.16 mm, *P* = 0.605) between the MTMC and PTMC groups. The incidences of a single nodule and lymph node metastases in the MTMC group (87.80 and 36.59%) were higher than those in the PTMC group (70.19 and 20.19%) (*P* < 0.05 for both).

**Table 1:** The clinical characteristics of MTMCs and PTMCs groups

Parameter	MTMC	PTMC	P
No. of nodules	46	136	
No. of patients	41	104	
Age(y)			
Mean	41.78 ± 16.39	44.07 ± 10.43	0.323
Range	14–80	24–71	
Gender			0.205
Male	18 (43.90%)	34 (32.69%)	
Female	23 (56.10%)	70 (67.31%)	
Size (mm)			
Mean	6.30 ± 2.27	6.06 ± 2.16	0.605
Range	2.0–9.91	1.5–10	
Multiplicity			0.027
Single	36 (87.80%)	73 (70.19%)	
Multifocal	5 (12.20%)	31 (29.81%)	
Lymph node metastases			0.040
None	26 (63.41%)	83 (79.81%)	
Present	15 (36.59%)	21 (20.19%)	

### Univariate analysis of US features

The results of the univariate analysis for US characteristics are summarized in Table 2. There were no significant differences in location, margin, peripheral halo ring or echogenicity between the MTMC and PTMC groups (*P* > 0.05 for all); however, significant differences were found in shape, boundary, composition, calcifications and vascularity between the two groups (*P* < 0.05 for all).

**Table 2:** The US characteristics of MTMCs and PTMCs on Univariate Analysis

Parameter	MTMC (*N* = 46)	PTMC (*N* = 136)	P
Location			0.86
Upper pole	24/52.17%	73/53.68%	
Lower pole	22/47.83%	63/46.32%	
Shape			0
Ovoid to round	25/54.35%	13/9.56%	0
Taller than wide	9/19.57%	81/59.56%	0
Irregular	12/26.09%	42/30.88%	0.538
Boundary			0
Clear	26/56.52%	19/13.97%	
Unclear	20/43.48%	117/86.03%	
Margin			0.101
Smooth	17/36.96%	37/27.21%	0.211
Spiculated	10/21.74%	53/38.97%	0.034
Poorly defined	19/41.30%	46/33.82%	0.36
Peripheral halo ring			0.595
Absent	41/95.65%	133/97.79%	
Present	2/4.35%	3/2.21%	
Echogenicity			0.727
Marked hypoechoic	10/21.74%	33/24.26%	
Hypoechoic	36/78.26%	103/75.74%	
Hyperechoic or isoechoic	0	0	
Composition			0.004
Solid	40/86.96%	134/98.53%	
> 50% solid	6/13.04%	2/1.47%	
Calcification			0.002
Absent	31/67.39%	87/63.97%	0.674
Microcalcification	5/10.87%	40/29.41%	0.012
Macrocalcification	10/21.74%	9/6.62%	0.004
Vascularity			0.000
Avascularity	19/41.30%	109/80.15%	
Hypervascularity	27/58.70%	27/19.85%	

### Multivariate logistic regression analysis of US features

**Fig. 1:**
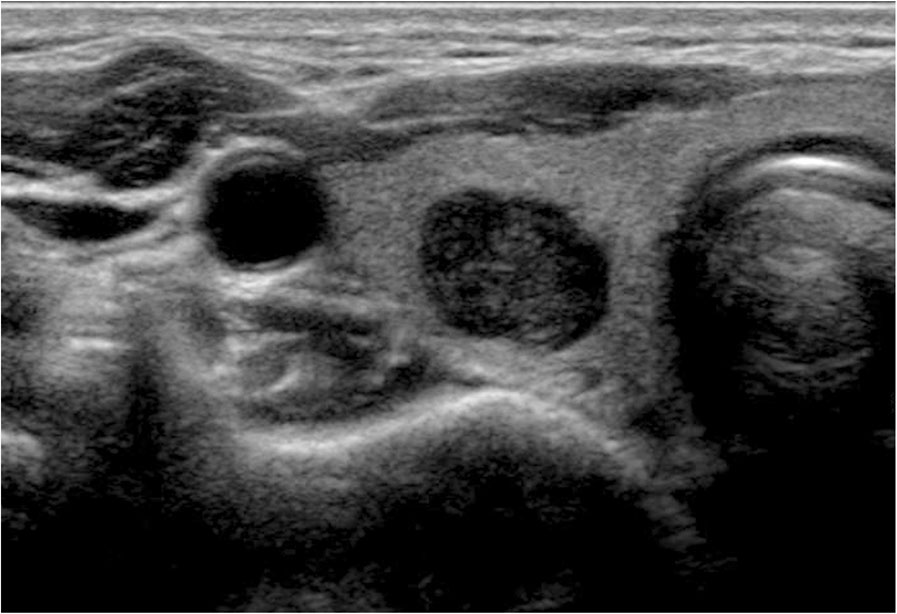
The sonographic features of ovoid to round shape, marked hypoechogenicity, solid composition, and smooth margin were shown on a transverse ultrasound image from a 40-year-old female with MTMC

**Fig. 2:**
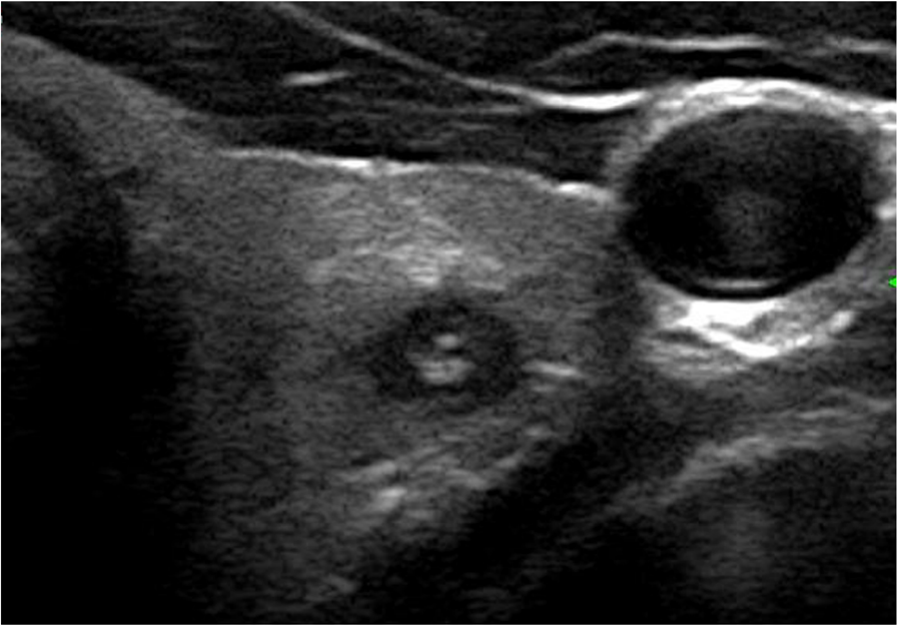
The transverse sonographic image of MTMC in a 68-year-old male showed hypoechogenicity, ovoid to round shape, and macrocalcifications

**Fig. 3:**
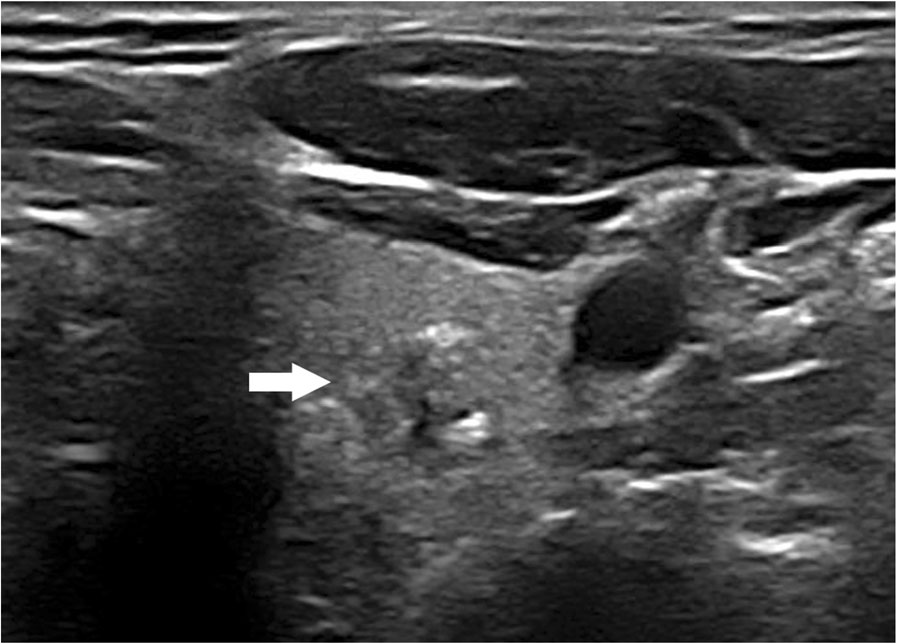
PTMC in a 36-year-old male. The transverse ultrasound image showed ovoid to round shape, unclear boundary and macrocalcifications

**Fig. 4:**
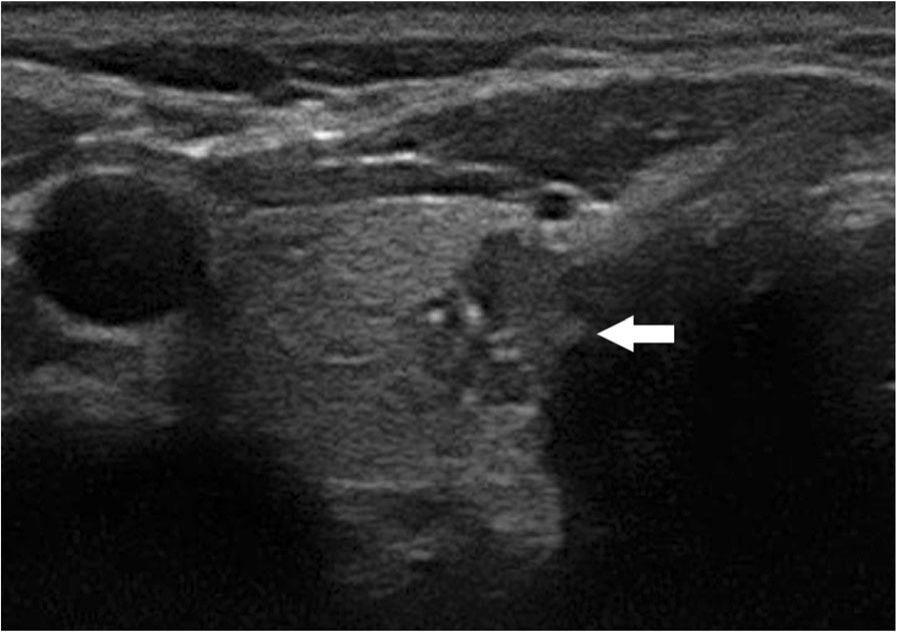
The PTMC transverse ultrasound image of a 28-year-old female showed taller than wide shape, poorly defined margin, and microcalcifications

**Fig. 5:**
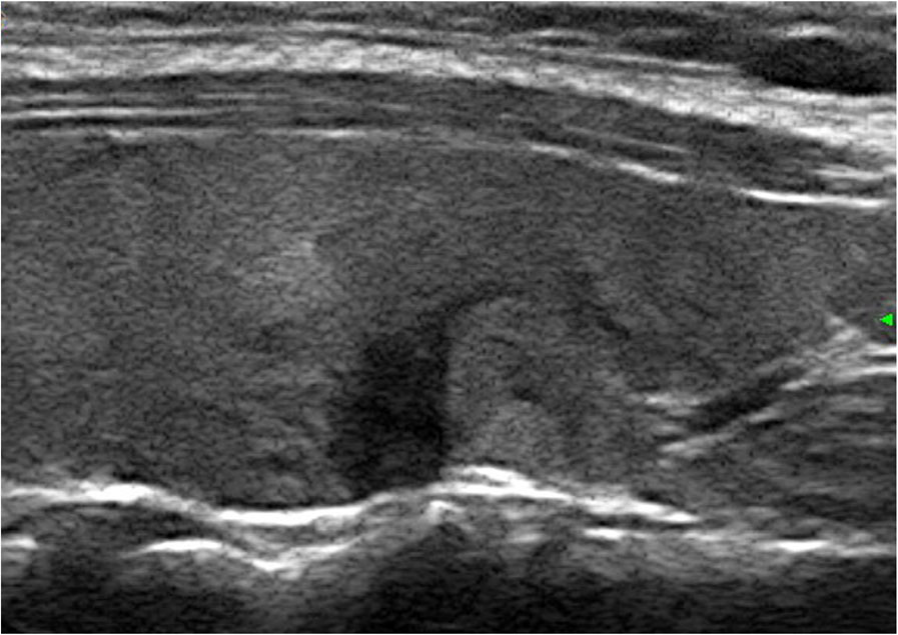
A 40-year-old female with PTMC. The longitudinal sonogram of the tumor presented a taller than wide shape and speculated margin

**Fig. 6:**
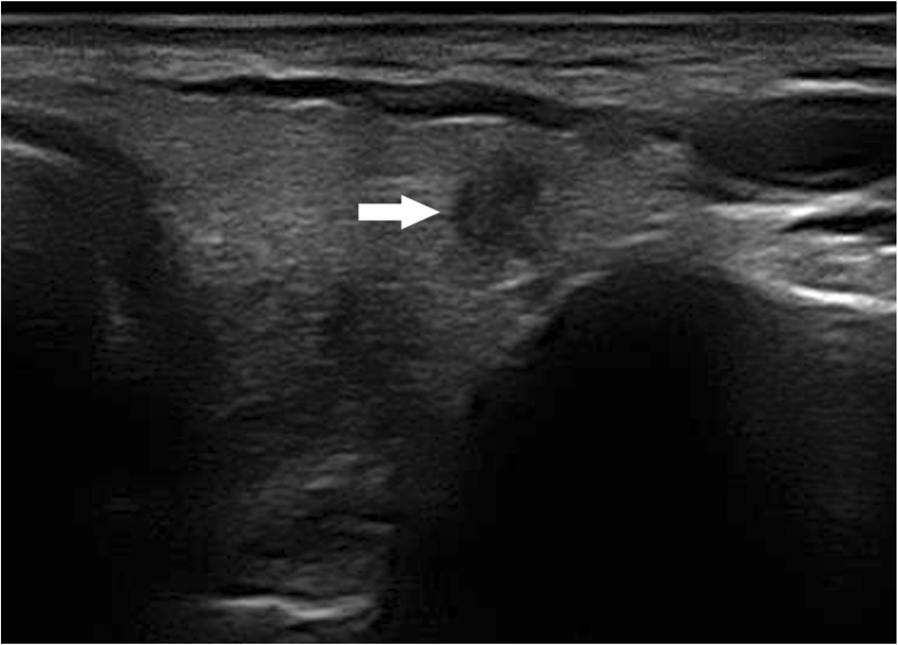
The MTMC nodule transverse sonographic image of a 76-year-old female displayed taller than wide shape, poorly defined margin, and hypoechogenicity

The results of the multivariate logistic regression analysis for US characteristics are summarized in Table 3. The proportion of MTMCs with ovoid to round shapes (Figs. 1 and 2) was higher than that of PTMCs (Fig. 3) (54.35% vs 9.56%, *P* = 0.007), but PTMCs with taller-than-wide shapes (Figs. 4 and 5) were much more common than MTMCs with the same shape (Fig. 6) (59.56% vs 19.57%, *P* = 0.000). An unclear boundary seemed to be more commonly detected in PTMCs (Fig. 3) than in MTMCs; however, there was no significant difference (86.03% vs 43.48%, *P* = 0.188). There were more nodules with a > 50% solid composition in the MTMC group than in the PTMC group (13.04% vs 1.47%, *P* = 0.032). Calcifications were not commonly observed in either group; when they occurred, macrocalcifications were more often seen in MTMCs (Fig. 2) than in PTMCs (Fig. 3) (21.74% vs 6.62%, *P* = 0.004). Microcalcifications were more frequently detected in PTMCs (Fig. 4), although there was no significant difference (10.87% vs 29.41%, *P* = 0.214). Hypervascularity was observed in 58.70% of MTMCs but only in 19.85% of PTMCs (*P* = 0.000).

**Table 3:** The US characteristics of MTMCs and PTMCs on Multivariate Logistic Regression Analysis

Variable	Multivariate Analysis				
	B	Wals χ^2^	Odds Ratio	95% CI	P
Shape		16.876			0
Ovoid to round vs Irregular	1.391	5.792	4.018	1.295–12.473	0.016
Taller than wide vs Irregular	2.602	16.869	13.488	3.897–46.681	0
Boundry	−0.623	1.735	0.536	0.212–1.356	0.188
Composition	−2.043	4.579	0.13	0.020–0.842	0.032
Calcification		8.808			0.012
Microcalcification vs absent	−0.829	1.543	0.436	0.118–1.615	0.214
Macrocalcification vs absent	−2.465	8.314	0.085	0.016–0.454	0.004
Vascularity	2.377	21.434	10.778	3.939–29.488	0

## Discussion

Previous studies have shown that MTC patients are more likely to be females, who have a single nodule in the majority of cases [9, 10]. In this study, there were no significant differences in age or tumor size between MTMCs and PTMCs. There was a slightly higher number of women than men among MTMC patients in this study; however, there was also no significant difference. In accordance with previous reports [9, 10], most patients with MTMCs had a single lesion, and only 5 patients had two MTMC nodules in the present study. PTCs often arise as multiple tumor foci, which are considered the result of multiple synchronous primary tumors arising from independent clones. The incidence of multifocal PTMCs has been reported as 23.5–36.1% [11–13]. In our study, multiple PTMCs were detected in 29.81% (31/104) of cases, which was higher than the corresponding percentage in the MTMC group. Our results showed that there was a significant difference in lymph node metastases between the MTMC group and the PTMC group. In the PTMC group, 21 (20.19%) patients had lymph node metastases, which is similar to the incidence in previous reports [14–16]. MTMC patients were more likely to have lymph node metastases, which indicates the strong invasive characteristics of MTCs. This also suggests that although the incidence of MTCs is very low, early diagnosis is of great significance.

Zhou et al. [7] found that MTMCs had no significant differences from PTMCs in internal composition, calcifications, echogenicity, margin, or shape (*P* > 0.05). In our study, there were no significant differences in location, margin, boundary, echogenicity, peripheral halo rings, or the presence of calcifications between the two groups (P > 0.05). However, there were significant differences in the following characteristics: internal composition, shape, calcification types and vascularity; these results are not exactly consistent with the results of Zhou. The possible reasons may be the different sample sizes, imaging instruments and evaluation standards of the ultrasonic characteristics.

Cystic changes are not common in PTMCs and MTMCs [7, 10]. Our results also showed that the majority of both MTMCs (86.96%) and PTMCs (98.53%) exhibited a solid composition; however, a > 50% solid composition was significantly more frequent in MTMCs than in PTMCs. In 46 MTMCs, a > 50% solid composition was observed in 6 (13.04%) lesions. Although the mechanism behind cystic changes is unclear, solid-cystic nodules might result from the degeneration of solid nodules, with the accumulation of fluid probably due to intranodular necrosis. MTCs tended to display less differentiation and grow faster than PTCs, and cystic changes in MTCs were most likely induced by necrosis as a result of cell proliferation that exceeded the available blood supply. Lee et al. [10] also reported that cystic changes were significantly more common in MTCs than in PTCs, and tumor size had no significant relationship with cystic changes.

PTC usually has highly intense fibrosis, and its compressibility is reduced, resulting in its upright morphology [17]. A taller-than-wide shape has been regarded as an ultrasonographic feature of PTMCs [18, 19]. In our study, this was also a common sign in the PTMC group, and the proportion of PTMCs with this sign was as high as 59.56%. However, there were only 9 (19.57%) tumors with a taller-than-wide shape in the MTMC group, which indicated that this sign did not apply to MTMCs. Previous studies have shown that, regardless of size, MTCs were mostly round or ovoid in shape rather than taller-than-wide [20, 21]. Similar results were also observed in our series. The MTMC nodules were more likely to be ovoid to round than PTMCs, and the difference was statistically significant (*P* = 0.000).

Calcifications, especially microcalcifications, have been regarded as a specific sign associated with thyroid malignancy. Kim et al. [22] found that the presence of calcifications was not significantly different between PTCs and MTCs. The results of our study showed that calcifications were not common in either the MTMC group or the PTMC group, and the incidence of calcifications was similar between groups (*P* = 0.674). However, the type of calcifications was different between the two groups. We found that microcalcifications were frequently seen in PTMCs, and macrocalcifications appeared more commonly in MTMCs. This might be related to the different mechanisms of calcification formation in MTCs and PTCs. Calcifications in MTCs are mainly due to the deposition of local calcium salts surrounded by amyloid substances, leading to the formation of coarse calcifications. However, calcifications in PTCs are mainly caused by psammoma bodies with a smaller diameter of 10 ~ 100 μm, which commonly appear round or concentric under a light microscope.

To the best of our knowledge, very limited literature has reported the blood supply of MTCs. Trimboli et al. [23] reported that the percentage of intranodular vascularization in MTCs (25%) was higher than that in PTCs (15%), but the difference was not significant. However, in the present study, MTMCs were more likely to show hypervascularity than PTMCs (*P* = 0.000). PTMCs were predominantly characterized by a reduced blood supply, which might be due to the incompletely developed neoangiogenetic vascular bed in comparison with the uncontrolled cell proliferation typical of carcinoma [24]. In the present series, 80.15% of PTMCs were deficient in vascularity. According to the study reported by Lai et al. [25], 72.4% of MTCs showed hypervascularity, which might be induced by the rapid division and growth of MTCs cells, resulting in a faster rate of proliferation than PTCs. In this study, an abundant blood supply was also commonly detected in MTMCs (58.70%), but the proportion was not as high as that reported in the literature. This may be due to the relatively small number of blood vessels caused by the small size of the tumor.

There were some limitations in our study. First, this was a retrospective study with statistical bias. Second, because of the low incidence of MTCs, it took a long time to collect MTMC cases. During this time, there have been significant developments in US technology, which are likely to have an impact on the evaluation of sonographic features. In addition, the sample size of the MTMC group was small, although there were more cases than in the previous studies. A further study with a larger sample size should be performed to identify the sonographic features of MTMCs.

## Conclusions

There were some overlapping sonographic features between MTMCs and PTMCs, and it was difficult to accurately distinguish between these two types of thyroid carcinomas. However, MTMCs were more likely to be ovoid to round nodules with a > 50% solid composition, have macrocalcifications and be hypervascular than PTMCs, and cervical lymph node metastases were more common in MTMC patients.

## Data Availability

All data in this study are available from the corresponding authors upon reasonable request.

## Abbreviations

MTC: medullary thyroid carcinoma
MTMC: medullary thyroid microcarcinoma
PTMC: papillary thyroid microcarcinoma
PTC: papillary thyroid carcinoma
US: ultrasound
MEN: multiple endocrine neoplasia
